# How Anxious are German Preschool Children?

**DOI:** 10.1007/s10578-021-01185-8

**Published:** 2021-05-08

**Authors:** Daniela Ehrenberg, Arnold Lohaus, Kerstin Konrad, Lorena Lüning, Nina Heinrichs

**Affiliations:** 1 Department of Psychology, Institute of Clinical Psychology, Psychotherapy and Assessment, University of Braunschweig, Humboldtstraße 33, 38106 Braunschweig, Germany; 2 Department of Psychology, Bielefeld University, Bielefeld, Germany; 3 Child Neuropsychology Section, Institute of Molecular Neuroscience and Neuroimaging (INM-11), RWTH Aachen University Hospital and Research Center Jülich, University Hospital Aachen, Juelich, Aachen, Germany; 4 Present Address: Department of Psychology, Clinical Psychology and Psychotherapy, University of Bremen, Bremen, Germany

**Keywords:** Anxiety, Preschool anxiety scale (PAS), Preschool children, Foster care

## Abstract

The experience of fear is universal and is among the earliest of all forms of psychopathology, if excessively present. To prevent negative developmental outcomes due to early-onset excessive fears in children, it is important to systematically assess these experiences as early as possible. Using the preschool anxiety scale (PAS), we aimed to assess the frequency and structure of anxiety symptoms of 489 preschool-aged children raised in their biological family and 88 raised in foster care (as a high-risk sample) in Germany. While these young children displayed the same types of anxiety most commonly as young children in other countries, the overall occurrence seems to be reported less often by parents in Germany compared to parents from other countries. Anxiety symptoms clustered into five correlated factors (generalized anxiety, social anxiety, obsessive–compulsive disorder (OCD), physical injury fear and separation anxiety). Young children in foster care exhibited more OCD and significantly less social anxiety symptoms indicating early repetitive and social disturbances in children in foster care.

**Supplementary Information:**

The online version contains supplementary material available at 10.1007/s10578-021-01185-8.

## Prevalence of Anxiety in Children and Adolescents

Anxiety disorders are the most frequent mental disorders in children and adolescents and can occur for the first time quite early in life (e.g., [1, 2]). Studies report prevalence rates of anxiety disorders in the kindergarten and preschool age between 10 and 20% (e.g., [1, 3]). This is similar to estimates in later developmental phases, including childhood and adolescence (15–20%) [1]. However, these rates vary considerably depending upon the country in which the data was collected, the method of measurement/informant (e.g. questionnaire/parent-report, structured clinical interview/clinician-rated) and (socio-) demographic factors (e.g. age, gender, socioeconomic status). For example, a study from Norway with 955 preschoolers reports a point prevalence rate of 1.5% anxiety disorders (assessed with the Preschool Age Psychiatric Assessment, PAPA) [4], whereas a current German study by Paulus, Backes, Sander, Weber, and von Gontard [5] with parents of 1342 children between 4 to 7 years identified 22% with high levels of anxiety (assessed with a 32-item parent report questionnaire with items drawn from the Child Behavior Checklist (CBCL), the DYSIPS-II and the Retrospective Infant Behavioral Inhibition Scale (RIBI)). Beesdo, Knappe and Pine [1] point out that those differences are unlikely reflecting true regional but rather methodological differences between studies.

Similarly controversial are studies regarding the association between fears or anxieties and age or gender. While studies with children and adolescents between 9 and 19 years show gender and age related differences regarding the occurrence of all mental disorders, including anxiety disorders (e.g. [6]), such studies with younger kids in the preschool age do not provide such a homogenous picture. Some studies discovered gender or age differences for some anxiety disorders (e.g. [5, 7–9]), others did not find significant age- or gender related differences (e.g. [7, 9–11]).

An important task in assessing fears and anxiety (disorders) is to decide if they are excessive in nature. The differentiation between normal and pathological experiences of fears can be especially difficult in young children when anxious experiences are a natural phase of normal development of children (e.g. [12, 13]). Separation anxiety can e.g. be seen as quite age-appropriate between 12 and 18 month as might be fears of thunder, lightning or darkness when they occur between age 2 to 4 (e.g. [1, 14]). Normal fears are often described as age-specific, mild, and temporary [15], however, this presumes specific data about the distribution of fears and anxiety across a certain developmental period. Bufferd, Dougherty and Olino [16] asked 291 primary caregivers of 3–5-year-old children to report the frequency of children’s daily separation and social anxiety behavior and related impairment for 14 days by using an innovative method based on diary. As a result, they were able to present dimensional information about the frequency and severity of anxiety behaviors (e.g., at which particular frequency is a behavior considered “psychometrically severe/rare”, p. 9) as well as the related impairment in early childhood. Results indicated that some behaviors were to be classified as rare by means of frequency (e.g. “worry that caregiver would not return”, p. 9) while others were not (e.g. “shyness around peers”). This study is a very helpful first step to evaluate if certain behaviors are normative (because many children show this behavior at a certain age) or not. However, contexts of anxiety and sub-group variations in children are identified as important further steps in research on preschool child anxiety. Thus, it is of uttermost importance to provide more detailed information on anxiety and fears in young children in order to facilitate the evaluation and interpretation of age-appropriate and clinically elevated symptom levels [17].

Looking at the developmental pathways of young anxious kids, there are homotypic (e.g. [18–21]) but also heterotypic progressions, for example with depression or oppositional defiant behavior (e.g. [8, 21]). Anxiety disorders in childhood seem to be a risk factor for depressive mood later in adolescence [22] and other psychopathological problems across the course of life (e.g. [23, 24]). In order to prevent negative effects of such early onset anxiety symptoms on children’s developmental pathways and therefore to detect maladjusted trajectories early in development, a reliable and valid assessment of such anxiety symptoms is needed before the age of 7 years [25–27].

## The Preschool Anxiety Scale (PAS)

The PAS provides an opportunity to assess fears and anxieties in this young age group [28]. The questionnaire was developed in 2001, is based on the DSM-IV classification system and involves 28 items summarized to five “anxiety disorder” symptom scales: social phobia, separation anxiety disorder (SAD), generalized anxiety disorder (GAD), obsessive–compulsive disorder (OCD) and physical injury fear (PiF) and six additional questions about post-traumatic stress disorder (PTSD) experiences and symptoms, answered by the parents. In light of the new DSM-5 (used since 2013), it is questionable whether the PAS is still reflecting these recent criteria. Looking at the differences between the DSM-IV and DSM-5 only minor discrepancies occur in how GAD and social phobia are defined. For SAD some formulations were changed (e.g. from “…to go to school” to “…to go out” and “social phobia” was renamed to “social anxiety disorder” (SA). However, the core criteria remained more or less the same (for an overview see [29]). The OCD criteria were not significantly changed although it was assigned to a different category in DSM-5 (e.g. [30]).

Unlike SA, SAD, GAD and OCD changes for PTSD between DSM-IV and -5 were significant. Similarly to OCD, PTSD is now part of a different category (“Traumatic- and Stress-Related-Disorders”). New criteria have been added for children aged 6 and younger being more developmentally appropriate for young children and focusing on behaviorally (for example through play) expressed PTSD Symptoms. The PAS does not reflect DSM-5 PTSD-related criteria and we will therefore not include the scale in the following paper.

Unlike the other anxiety scales, the PAS—“physical injury fear” scale was never part of the DSM. Spence et al. included this scale as it summarizes multiple items relating to different specific phobias [28]. In addition, the authors found a strong fear of physical injury in older children ([31, 32]). The existence of such fears has also been reported by other authors (e.g. [12, 33]). In sum, the PAS provides information about the type and frequency of anxiety symptoms and evaluates whether a child is at risk for developing anxiety disorders. While the questionnaire was originally developed based on the DSM-IV, many of the scales may hold up to the DSM-5 except for the PTSD scale (for more information on the PAS see www.scaswebsite.com).

Since the development of the PAS in Australia, the questionnaire has been evaluated [34–41] and used in anxiety research (e.g. [42–45]) across many different countries. The studies used either a translation of the PAS or the revised version PAS-R from Edwards, Rapee, Kennedy and Spence with less items and a four-factor model (social anxiety, generalized anxiety, separation anxiety, specific fears) [7]. Mean total and subscale scores across countries sometimes seem to be quite similar, for example for the 3-year olds [28, 34, 35] but also differ considerably at times (e.g., the mean value of the PAS total score for the Portuguese 5-year olds [34] was nearly twofold compared to the Australian children [28]).

The PAS with its multiple investigations across countries offers an opportunity to reflect parent reported anxiety symptoms of preschool children in different parts of the world. A recent meta-analysis mentioned the importance of identifying prevalence estimates variability for example in order to address questions about etiology [2].

All studies using the PAS found age related differences, but the direction of these differences varied across studies. While Spence et al. [28] and Wang and Zhao [40] found higher scores on all scales for the younger children, Benga et al. [35] and Broeren and Muris [36] solely found the older children to score significantly higher on the SA and the GAD scale. Almeida and Viana [34] reported a positive correlation between age and fears. With regard to gender, some PAS studies found no difference [28, 34, 40] whereas Broeren and Muris [36] report significantly and Benga et al. [35] a tendency towards higher scores for girls.

Although age and gender differences across countries vary, factorial validity seems rather stable: the assumed five correlated factor structure was supported in many studies (except for Benga et al. [35]) in preschoolers [28, 34, 36–41]. Other models that have been discussed are related to a single factor model, assuming that all items load onto one single anxiety factor, without differentiating between different clusters of anxiety. This model would prevail over the other models if parents perceived anxiety in their children as one phenomenon rather than as distinct clusters of symptoms. Although this model was not holding up well in former PAS studies, there are indications that a higher-order factor model may provide a good explanation for the high correlations between the five factors [28, 31, 35] and for other anxiety factors identified in studies using other instruments than the PAS [33, 46].

In light of a lack of data for anxiety during the age range of 2–6 years in Germany, we aim at (1) examining the means, standard deviations, and internal consistencies of the PAS in young children between the age of 2–6 years living in Germany, (2) exploring the frequency of anxiety symptoms in these young children, (3) determining the proportion of children who would require further assessment to determine the presence of an anxiety disorder, (4) comparing mean PAS scores of this sample from Germany to previously reported PAS data from other countries and finally (5) determining the factor structure assuming that the five correlated factor model will also hold up best in this sample from Germany.

In addition to a sample recruited via preschools, a high-risk sample of children living in foster care was included in the current study. Children living in foster care often experienced various forms of early adversity (such as maltreatment, neglect, loss and separation experiences) before the out-of-home placement [47] increasing the risk for developing anxiety or depression later in life [48]. A recent meta-analysis including 96 studies examining different forms of child maltreatment (defined as any form of sexual, physical or emotional abuse as well as neglect and exposure to interpersonal violence before the age of 18) showed that several forms of child maltreatment were associated with different anxiety disorders later on in life [48]. Thus, in the current study, aims 1–3 (investigating PAS scores distribution, reliability, and interpretation of clinically relevant scores) were further investigated in children living in foster care expecting overall increased number of anxious symptoms in this high-risk group.

## Methods

### Participants

This study is based on data from two projects, one assessed fears in preschoolers (among other constructs) as part of a larger project with the aim of monitoring the development of young children (with or without substantiated maltreatment experiences), and the other one specifically focused on assessing fears in preschoolers with the PAS.

The first study (GROW&TREAT) focused on children (2–6 years) who were exposed to early childhood adversities which led to an out-of-home placement into a foster family for part of the sample while the other children were not exposed to such adversities and were raised in their biological families (study 1). The study was conducted between 2013 and 2016 in three different north western regions in Germany. The sample consisted of 94 children living in foster and 157 children living in their biological families (e.g. [49–51]). In total, 251 parents (mostly mothers) reported about their child (in foster care) completing the PAS. In terms of the children’s fears, we expected mean differences (i.e. different mean levels of fears) with children in foster care being more anxious due to their (family-related) disruptive experiences in the past, however, we did not expect structural differences between the fear assessments.

The second study was conducted to additionally recruit a more representative sample of parents with preschool children in order to validate the PAS (study 2). In total, 354 parents (mostly mothers) participated in this cross-sectional project. The data was collected between June and October 2018 in 20 preschools (including nurseries for 2-year olds) in a region very close to one of those participating in study 1. Depending on the structure and needs of the preschools, a total of 1500 PAS questionnaires were provided directly to the parents, placed in the compartments of each child for the parent or displayed on a clearly visible place for take away. Questionnaires were returned anonymously into sealed boxes displayed in each participating kindergarten [52].

The prerequisite for participating in these projects was to fit into the age range between 2;0 and 6;11 years. As a result, 22 children (12 from study 1 and 10 from study 2) had to be excluded. Six children of study 1 could furthermore not be included because their parents did not complete the PAS questionnaire. The final sample (including 88 children in foster care) consisted of 233 children from study 1 and 344 children from study 2.

### Instruments

*Preschool Anxiety Scale (PAS)*: The Preschool Anxiety Scale [28] is used to assess different dimensions of anxiety in preschool children. It was developed on the basis of the Spence Children’s Anxiety Scale (SCAS; [31]). The questionnaire includes five subscales: generalized anxiety disorder (GAD), social anxiety (SA), obsessive–compulsive disorder (OCD), physical injury fears (PiF) and separation anxiety disorder (SAD). Parents indicated for 28 items their estimation about the anxiety of their children using a five-point scale, ranging from 0 (“Not True at All”) to 4 (“Very Often True”). According to the authors of the PAS a symptom of anxiety can be seen as “present” if parents rated the related item with 3 (“Quite Often True”) or 4 (“Very Often True”). PAS total score (TS) and subscale scores can be calculated by summing across relevant items. There are six additional questions about post-traumatic stress symptoms which are not included in the total score. A larger score means that parents perceive anxieties in their children more frequently.

### Data Analysis

The software IBM SPSS Statistics for Windows, Version 25.0 was used to analyze the data. *p*-values of < 0.05 were considered as significant. Furthermore, the confirmatory factor analysis was conducted using IBM SPSS Amos, Version 25.0. RMSEA values below 0.05 were evaluated as good, between 0.05 and 0.08 as adequate and above 0.08 as an indicator for bad model fitting [53, 54]. For the NFI, CFI and TLI we specified values of at least 0.90 as an indicator for a good model fit [53, 55, 56]. The model chi-square (*χ*^2^) *p*-value should ideally exceed 0.05 [53].

If necessary for the analysis, children were categorized into the following age groups: 2-year olds (24–35 months), 3-year olds (36–47 months), 4-year olds (48–59 months), 5-year olds (60–71 months) and 6-year olds (72–83 months). All *p*-values are reported two-sided. We will first check if the two study samples may be merged (preliminary analysis) before we report results along the primary aims of the study.

### Missing Values

The evaluation of the missing values of the PAS by means of Little’s MCAR test showed that missings were not completely at random (MCAR), *p* < 0.05. After excluding children with unexpectedly high numbers of missing items (e.g. an entire page of the questionnaire)^1^ Little’s MCAR test still showed significant results (*p* < 0.05). Out of 15,925 possible item answers, 91 (1%) were missing. All 91 values were distributed over 49 cases making up approximately 9% of the samples. In face of such a low proportion of missing item responses, Expectation Maximization (EM) was used for imputing missing values. The most frequently missing item (n = 12, 2%) was item 25 “Has nightmares about being apart from you” followed by item 15 “Is afraid of talking in front of the class (preschool group) e.g., show and tell.” (n = 9, 2%).

## Results

### Preliminary Analysis

The two studies included three samples drawn from the population of children aged 2–6 years: two independently drawn samples of children raised in their biological families and one sample of children raised in foster care. As we assumed the kids in foster care to reflect a different population, this group was handled separately from the others. In contrast, we assumed the other two samples to be part of the same population*:* they were recruited very similarly (although for different purposes and these may also require a different level of parental engagement) and in similar regions. When comparing these two samples on sociodemographic variables (age and gender of child, gender of informant), no differences occurred between these two samples (*age:* mean of 52 months (*SD* = 16.56) in study 1 and 51 months (*SD* = 13.04) in study 2*, t*(229.51) = 0.85, *p* = 0.40; *gender*: 49% females in study 1 and 47% in study 2*, χ*^2^(1, *N* = 489) = 1.03, *p* = 0.31). In study 1, 10% of the questionnaires were filled out by mother and father together, in study 2 only 3%. Whether the questionnaire was completed by one caregiver alone or two caregivers together was therefore significantly different (*χ*^2^(1, *N* = 484) = 11.34, *p* = 0.001). Nonetheless, there was no significant difference concerning the distribution of mothers and fathers completing the questionnaire alone over the two samples (mothers: 78%, fathers: 10% and 87% and 9%, respectively, *χ*^2^(1, *N* = 459) = 0.46, *p* = 0.50). Based on the analysis we decided to combine the two samples of children raised in their biological families for further analyses.

### Sample Description

For the total sample of children raised in their biological families (*N* = 489) a similar proportion of girls (n = 239) and boys (n = 250) occurred. 35% of the mothers indicated a university degree, 24% completed 13 years, 25% 10 years and 4% 9 years of school education. Another 1% had other school-leaving qualifications and 1% missing values. On the other side, 34% of the fathers had a university degree, 23% completed 13 school educational years, 22% 10 educational years and 8% 9 educational years. In addition to that, 3% named other school-leaving qualifications. Missing values made up 10% for this variable.

The sample of children living in foster care included 43 girls and 45 boys. The children were on average 43 months old (*SD* = 15.62). There was no significant age difference between boys and girls (*t*(86) =  − 0.20, *p* = 0.84). Children living in their biological families were significantly older than those living in foster care (*t*(565) = -4.89, *p* < 0.001). There was no difference in gender distribution (*χ*^2^(1, *N* = 577) = 0.00, *p* = 1.00) between the two samples (children living in foster care versus in their biological families).

### Main Analyses

#### Outliers

When looking at the distribution of values of the PAS it can be seen that the total score and all subscales showed a left steep or rather right skewed and sharp distribution, with GAD and OCD scales leading. With the exception of the SA scale, outliers with values exceeding three standard deviations can be seen on all scales. All outliers could be assigned to nine parents. It was not possible to detect a reason for the striking high values with the help of the raised data. Apparently, the nine parents were not different from the others and as a result none of them was excluded. For further details about the distribution of values see Table E-1 in the electronical supplements.

#### Objective 1: Means, SD and Reliability of the PAS

For children raised in biological families Cronbach’s alpha of the total sore and the scales GAD and SA varied between 0.74 and 0.85 and can therefore be seen as acceptable to good. On the other hand, values of the scales OCD (*α* = 0.48), PiF (*α* = 0.57) and SAD (*α* = 0.56) were in the low to moderate range (e.g. [57]). For children raised in foster care Cronbach’s alpha of the total sore and the scales GAD, SA and SAD varied between 0.73 and 0.88.

As for children living in biological families, the scale PiF (*α* = 0.52) was in the low to moderate range.

Children living in foster care showed a significantly (*t*(575) =  − 2.65, *p* < 0.01) lower score on the social anxiety scale (Table 1). There was a significant difference (*p* < 0.05) between boys and girls growing up in their biological families on the TS (*t*(487) =  − 2.12) and the scales PiF (*t*(487) =  − 2.41) and SAD (*t*(487) =  − 2.02) with girls scoring higher than boys on these scales. There was no significant difference between boys and girls living in foster families (Table E-2).

**Table 1:** Means, standard deviation, internal consistency and *p*-value for between-group differences in means for child anxiety separated by biological and foster parent ratings

	Biological parents^a^			Foster parents^b^			
	*M*	*SD*	*α*	*M*	*SD*	*α*	*p*
TS (28 items)	14.98	10.36	.852	14.70	12.14	.878	.821
GAD (5 items)	1.71	2.25	.741	2.18	2.90	.740	.146
SA (6 items)	4.04	3.66	.780	2.93	3.21	.752	.008**
OCD (5 items)	1.06	1.78	.483	1.53	2.13	.358	.050
PiF (7 items)	5.15	3.75	.566	5.00	3.91	.526	.713
SAD (5 items)	3.04	2.75	.563	3.07	3.49	.731	.936

*TS* total score of the PAS, *GAD* generalized anxiety disorder, *SA* social anxiety, *OCD* obsessive–compulsive disorder, *PIF* physical injury fears, *SAD* separation anxiety disorder

^a^*N* = 489, ^b^*N* = 88, ** *p* < .01

Post-hoc correlative analysis of the relationship between time in foster care (in months, *M* = 17.35, *SD* = 8.89) and each of the PAS scales was conducted. None of the correlations was significant (*p* between 0.20 (OCD) and 0.90 (PAS total score)) with correlation coefficients ranging from − 0.14 (OCD) to 0.06 (SA) indicating no statistical associations between time in foster care and the PAS scales.

#### Objective 2: Frequency of Anxiety Symptoms

The frequency of the six most frequently mentioned anxiety symptoms in percentage can be seen in Table 2. All other items were perceived by less than 7% of the parents as quite often or very often true for their child. A complete overview of all answers can be seen in Table E-3. The most frequently mentioned item was item 6 “Is reluctant to go to sleep without you or to sleep away from home”.

**Table 2:** Rank order percentage of children receiving parent ratings of 3 or 4 (Quite Often True and Very Often True) for the six most frequently mentioned items for biological (BF) and children raised in foster care (FC)

		% Of ratings ≥ 3	
	PAS Items	BF	FC
6	Is reluctant to go to sleep without you or to sleep away from home	20.5	20.4
20	Is afraid of insects and/or spiders	12.1	17.0
13	Is scared of thunder storms	10.6	10.2
11	Is afraid of meeting or talking to unfamiliar people	10.5	7.9
24	Is frightened of dogs	10.2	4.5
26	Is afraid of the dark	9.4	18.2

*BF* children raised in their biological families, *N* = 489; *FC* children raised in foster care, *N* = 88

#### Objective 3: Interpretation of Scores

Spence offers means and standard deviations separated by age and gender for every PAS scale (see www.scaswebsite.com). For the interpretation it is suggested that a score of 1 standard deviation above the mean for a subscale or total score “would warrant further investigation” whereas a score above 0.5 standard deviations above the mean score is “indicative of an elevated, but not clinical level of anxiety”. Table 3 shows the range of values accounting for a “normal” and “elevated but not clinical” level of anxiety and the cut-off for interpreting the score as “would warrant further investigation”. On average, across scales 10% showed elevated and 17% “to be further investigated” scores. Numbers were a little bit higher for children living in foster care (10% and 19%). There was a significant difference between the children raised in their biological and those raised in foster families on the scale OCD with the latter assigning proportionally more children in the elevated and warrant further investigation group (*χ*^2^(2, *N* = 577) = 9.99, *p* < 0.01).

**Table 3:** Percentages and total number of elevated anxiety expression that warrant further investigation across samples when applying the German norm values

	Range			Sample of children raised in their biological families^a^			Sample of children raised in foster families^b^		
	Normal	Elevated	Further investigation	Normal % (n)	Elevated % (n)	F. invest. % (n)	Normal % (n)	Elevated % (n)	F. invest. % (n)
TS	0 − 19	20 − 24	25 +	70 (344)	12 (60)	17 (85)	72 (63)	13 (11)	16 (14)
GAD	0 − 2	3	4 +	74 (362)	10 (49)	16 (78)	67 (59)	9 (8)	24 (21)
SA	0 − 5	6 − 7	8 +	72 (351)	12 (59)	16 (79)	80 (70)	11 (10)	9 (8)
OCD	0 − 1	2	3 +	75 (365)	12 (56)	14 (68)	59 (52)	15 (13)	26 (23)**
PiF	0 − 7	8	9 +	77 (375)	5 (25)	18 (89)	77 (68)	6 (5)	17 (15)
SAD	0 − 4	5	6 +	74 (362)	10 (51)	16 (76)	74 (65)	5 (4)	22 (19)

*TS* total score of the PAS, *GAD* generalized anxiety disorder, *SA* social anxiety, *OCD* obsessive–compulsive disorder, *PIF* physical injury fears, *SAD* separation anxiety disorder, *Normal* values lower than 0.5 standard deviations above the mean score, *Elevated* between 0.5 and 1 standard deviations above the mean score indicates “an elevated, but not clinical level of anxiety”, *F. invest* a score of 1 standard deviation or more above the mean for a subscale or total score is interpreted as “would warrant further investigation”

^a^*N* = 489, ^b^*N* = 88

^**^p < .01

#### Objective 4: Means and Standard Deviations for Each Age Group Compared to Other Internationally Published Studies on the PAS

When comparing the mean values of the German sample to those from other countries it can be seen that the German children score overall the lowest (Table 4) with effect sizes varying from insignificant (0.03) to very large (1.52; Table E-4). On the SA scale German children between the age 2 to 4 and 5 to 6 were more anxious than those coming from the Netherlands (*d*_2-4_ = 0.27 and *d*_5-6_ = 0.29) and those coming from Australia at the age of 5 (*d* = 0.27). Overall effect sizes were small for these three values. For all other scales and ages effect sizes varied from insignificant (*d*_SAD,Ger-Aus,4 years_ = 0.03) to very large (*d*_OCD,Ger-Rom,3 years_ = 1.52) with the German children scoring lower in every case. Concrete effect sizes for every PAS scale, age and country are reported in Table E-4.

**Table 4:** Means and standard deviations (in parenthesis) of the present sample and the samples of other PAS studies grouped into countries and age

Age	2	3				4				2–4	5				5–6	6
	Ger	Ger	Aus	Rom	Port	Ger	Aus	Rom	Port	Neth	Ger	Aus	Rom	Port	Neth	Ger
N	76	118	270	118	?	144	402	200	?	137	122	551	236	?	135	29
TS	12.5 (11.9)	14.6 (9.3)	22.9 (15.6)	29.2 (14.9)	28.1 (12.8)	14.9 (9.3)	18.8 (13.9)	31.6 (15.1)	29.3 (14.0)	18.0 (11.9)	16.9 (11.5)	18.3 (12.2)	30.6 (14.8)	34.8 (15.9)	19.5 (11.2)	15.6 (9.0)
GAD	1.3 (2.4)	1.7 (2.1)	3.1 (3.3)	4.1 (3.1)	4.9 (3.2)	1.4 (2.0)	2.5 (3.1)	4.3 (3.5)	4.7 (3.0)	2.5 (2.8)	2.3 (2.6)	2.8 (2.8)	4.1 (3.1)	5.7 (3.5)	3.3 (3.0)	2.2 (2.0)
SA	2.8 (3.3)	3.8 (3.0)	6.0 (5.4)	5.2 (4.2)	6.1 (3.2)	4.2 (3.9)	5.4 (4.9)	6.5 (4.7)	6.5 (3.8)	4.7 (4.2)	4.7 (4.1)	5.4 (4.2)	6.1 (4.3)	7.8 (4.3)	6.1 (4.1)	4.6 (3.1)
OCD	1.2 (2.4)	1.1 (1.7)	1.9 (2.6)	5.0 (3.2)	3.8 (3.0)	1.0 (1.7)	1.1 (1.9)	4.9 (3.1)	4.6 (3.2)	1.6 (2.3)	1.1 (1.7)	1.3 (1.8)	4.9 (3.2)	4.5 (2.9)	1.2 (2.1)	0.7 (1.1)
PiF	4.3 (4.0)	5.0 (4.0)	8.0 (4.9)	8.8 (5.7)	7.5 (4.5)	5.3 (3.3)	6.7 (4.7)	9.9 (5.6)	8.8 (5.2)	6.9 (4.5)	5.7 (3.8)	6.0 (4.4)	9.8 (3.5)	10.7 (6.1)	6.5 (3.7)	5.1 (3.5)
SAD	2.9 (2.9)	3.1 (2.6)	3.9 (3.6)	6.2 (3.6)	6.1 (3.9)	3.0 (2.6)	3.1 (3.3)	6.0 (3.5)	6.2 (3.9)	2.3 (2.4)	3.2 (3.0)	2.8 (2.8)	5.7 (4.0)	7.3 (4.4)	2.3 (2.4)	3.0 (2.8)

*Ger.* Germany, the present study, *Aus.* Australia, Spence et al. (2001), *Rom.* Romania, Benga et al. (2010), *Neth.* Netherlands Broeren & Muris (2008), *Port.* Portugal, Almeida & Viana (2013). In the Portuguese sample it is not given how the number of children spreads across the age groups (*N* = 562, *M* = 51.19 month, *SD* = 13.39). *TS* total score of the PAS, *GAD*  generalized anxiety disorder, *SA* social anxiety, *OCD* obsessive–compulsive disorder, *PIF* physical injury fears, *SAD* separation anxiety disorder

#### Objective 5: Psychometric Properties of the PAS

In terms of the children’s fears, we expected mean differences (i.e. different mean levels of fears) with children in foster care being more anxious due to their (family-related) disruptive experiences in the past, however, we did not expect structural differences in fears. As a result, we decided to merge the two samples of children living in their biological families and those living in foster care for the following confirmatory factor analysis.

#### Confirmatory Factor Analysis

We tested whether preschool anxiety symptoms could be better explained with a single general anxiety dimension or with a five factor model. The first model (Model 1) would suggest that symptoms of anxiety in preschool children reflect a single dimension of anxiety, on which all items loaded strongly, with minimal variance left to be explained by separate anxiety disorder factors. The second model is based on the DSM-IV categories of anxiety (Model 2). It presumes that the examined anxiety symptoms cluster into five correlated dimensions (social anxiety, separation anxiety, generalized anxiety, obsessive–compulsive disorder and physical injury fear). The third model (Model 3) can be seen as a combination of Model 1 and Model 2. As in Model 3 items are proposed to cluster into five factors but with an additional higher-order factor standing for a construct of general anxiety. This model can be seen as in line with the DSM-IV as well. The three models were compared to each other to determine which model provided the most adequate fit of the data.

#### Model 1: Single Factor

Confirmatory factor analysis using the one factor model revealed that 18 of the 28 items showed a loading exceeding 0.40 on the single factor, and another six items loading with > 0.30 but < 0.40. Four items had a loading of < 0.30 with the item “Is frightened of dogs” presenting the lowest loading (0.17). Overall the single factor solution is not a good fit of the data, with NFI, TLI and CFI statistics being < 0.90, RMSEA values exceeding 0.08 and the *χ*^2^ statistic being statistically significant (Table 5).

**Table 5:** Fit indices and χ^2^ statistics of the three investigated models displaying possible structures of preschool anxieties

Model	*χ*^2^	df	*p*	NFI	CFI	TLI	RMSA
Model 1 one factor	1884	350	.000	.585	.631	.601	.087
Model 2 five correlated factors	1295	340	.000	.714	.770	.744	.070
Model 3 five first-, one second-order factor	1329	345	.000	.707	.763	.741	.070

#### Model 2: Five Correlated Factors

For the five-factor model each item was forced to load uniquely on its hypothesized dimension, with factors being allowed to inter-correlate. As shown in Table 5, the NFI, TLI and CFI values did not exceed 0.90, with the RMSEA value around 0.07. The *χ*^2^ results displayed a significant difference between the parameters of the data and the model *χ*^2^ (340) = 1295, *p* < 0.001. The change in the χ^2^ statistic in relation to change in degrees of freedom between the five-correlated factor model and the one factor model indicates a significantly better fit of the data by the five-factor model (*χ*^2^Δ = 588, dfΔ = 10, *p* < 0.001). Thus, the five-factor model was taken as the preferred model for further examination of the data. The factor loadings of each item upon its hypothesized factor are shown in Table E-5 and exceeded 0.40 in 24 of 28 cases. The five factors were found to be strongly inter-correlated, with all values exceeding 0.50 (Table E-6).

#### Model 3: Five Factors Loading Onto One Higher-Order Factor

A higher-order model was examined to determine whether the high level of covariation between the five anxiety factors could be accounted for by a higher-order factor of “anxiety”. As Table 5 indicates, Model 3 did not provide a good fit of data with NFI, CFI and TLI all under 0.90 and a RMSEA value above 0.05. The standardized loadings of the first order factors upon the higher-order factor were all high, being 0.89 for the GAD factor, 0.56 for SA, 0.94 for OCD, 0.75 for PiF and 0.93 for SAD. In order to determine the extent to which the covariation between the first order factors can be accounted for by a higher-order factor the value of the target coefficient as described by Marsh and Hocevar [58] was calculated. The comparison of the *χ*^2^ values of Model 2 and higher-order Model 3 produced a target coefficient of 0.97, suggesting that the higher-order model provides a satisfactory explanation for the covariance between first order factors [58].

## Discussion

Overall, our analyses revealed that the German version of the PAS has good psychometric properties. Nevertheless, unexpected results showed up when comparing children living in their biological families and those living in foster care. We expected foster children to show more anxious behavior and as a result to score higher due to their prior experiences in their family of origin but this was only supported for OCD (*p* = 0.05). A clearly significant difference on the social anxiety scale occurred, however, with children in foster families scoring lower (and not higher) on this scale. We suppose that some foster children may develop a disinhibited social engagement disorder or symptoms of indiscriminately friendliness due to experienced neglect in the first two years of life (e.g. [59]). As children with such symptoms actively approach and interact with unfamiliar adults it is probable that they are rated as socially fearless by their caregivers. Further studies should investigate this topic to identify possible explanations for reduced social anxiety symptoms in children living in foster care. Additionally, later analyses of the cut-offs also showed that significantly more children living in foster families showed conspicuous compulsive behavior. Several new studies have shown that there is a connection between OCD and the experience of maltreatment in the childhood [60, 61]. To give an example, a new Canadian health survey with 25097 participants has shown that 72% of people with OCD hat experienced some form of childhood maltreatment [61]. Further studies should investigate whether the PAS is an adequate instrument to detect conspicuous compulsive behavior in children living in foster care. Furthermore, research on the developmental course of anxiety in children in foster care would be interesting. In a longitudinal study with 154 children with and 88 children without the experience of maltreatment before the age of 4 assessed anxious and depressive symptoms by using the CBCL every two years until the age of 10. The study showed that at age 4 there was no significant difference in anxiety/depression between the two groups. However, children with maltreatment experience showed a significantly greater increase in symptoms over the following six years than children without such a history [62] illustrating the need to carefully consider the developmental course of anxiety in high-risk samples. This study suggests that differences in anxiety between children with and without maltreatment experiences increase over time and become apparent by age 10 [62]. It is possible that the children in the present sample were too young to detect significant between-group differences and that differences in anxiety disorders between children with and without maltreatment experiences might only emerge at a later age.

The most frequently approved item from both groups of children was item 6 “Is reluctant to go to sleep without you or to sleep away from home”. This result is congruent to the Portuguese PAS study from Almeida and Viana [34]. Overall, parents in the present study appointed the same six items as most frequently occurring compared to the study by Spence et al. [28]. Simply the order between these six items differed slightly. The same results occurred for the least named items. The content of the most or least frequently experienced anxieties seems to be similar when comparing kids living in Germany with those from other countries, the intensity (i.e. mean levels) seems, however, to differ between all scales and ages. The means were closest to those from Australia and the Netherlands (*d* between 0.03 and 0.65) whereas compared to Romania and Portugal values were half in size (*d* between 0.33 and 1.52). One explanation for the comparably high values in these countries may be the poorer economic situation (e.g. [63, 64]) especially, when the global economic crisis in 2008/ 2009 is considered which hit poorer countries especially hard and made their economic situation even worse [65]. One could suspect that parents may have transferred their (existential) anxieties to their children. Romania is still one of the three financially poorest countries in the EU [63] whereas Portugal is constantly growing economically [64]. It would be interesting to investigate anxieties of Portuguese preschoolers in a few years again to see whether the improving economic situation may have resulted in lower anxiety levels now compared to 2012. Overall some former studies have shown that a low income and low socioeconomic status is closely connected to many emotional and behavioral disorders including anxiety disorders (e.g. [66, 67]). Nonetheless, these are only suggestions that need to be further investigated. In addition to that, one limitation of all PAS studies is that there was no testing for measurement invariance. This aspect should be addressed in future studies.

We applied the rule of the Australian standardization by using the mean score and adding 0.5 or 1 standard deviation to define “elevated” or “would warrant further investigation” recommendation to our sample. As a consequence, on average across all scales 17% of the children living in their biological families and 19% of the children living in foster care showed values that would warrant further investigation which corresponds with the frequency of anxieties in previous studies. Beyond that, it is noticeable that by removing the social anxiety scale (where children living in foster care scored particularly low) the average percentage of conspicuous values for children living in foster care rises to 21%. This may be an indication that although there was no significant difference in mean values when comparing the two groups of children, more children living in foster care may be at risk to develop anxiety disorders.

The present results suggest that the PAS may be an appropriate instrument to screen for anxiety and fears in young children. Nevertheless, it is crucial to add structured interviews in further studies to determine the number of children with an actual anxiety disorder and match those results to the related PAS scores. In their study, Bufferd and colleagues identified a large range of normative behavior based on parents’ 14-day diary report. This is an interesting approach to capture the large variation in frequency and severity of anxiety behaviors in young children. It demonstrated that only 1 of 4 behaviors for social anxiety and 4 of 8 behaviors in separation anxiety were classified as problematic (less normative). Interestingly, parents of young children reported more social anxiety than parents of older children, again supporting the hypothesis that lack of age-typical social anxiety may be of particular importance in children with maltreatment experiences [16]. It should be taken into account that Spence et al. [28] included the 2-year olds in the evaluations of psychometric properties and the factor analysis but not in the symptom scores and the presentation of frequency rates based on cut-off scores. These age differences may lead to lower mean values in the present German compared to the Australian sample. Unlike Spence et al. [28] we found the youngest children scoring lower on all scales compared to the other age groups. One of Spence et al.’s explanation is that in Australia most of the children start preschool at the age of 3 [28, 68] and the elevated frequency of anxiety symptoms may be a response to this life transition. The influence of developmental transitions on anxieties in children has already be discussed in former studies (e.g. [69]). In Germany, many children already attain some kind of preschool care under the age of 3 years with numbers constantly rising [70]. As most of the data for the 2-year olds in this study were collected in kindergartens it is highly probable that they were just getting used to daytime care whereas many of the 3-year olds had already time to adapt. This can be one explanation for the great difference between the 3-year olds from our study compared to those from other PAS studies. The question remains why the 2-year olds in our study show the lowest values although they face the transition to daytime care. The most likely explanation is that the 2-year olds were too young to show all the behavior or express the thoughts inquired by the PAS. Some parents noted beside the questionnaire that they were not able to evaluate some items because their child was too young to express such thoughts or show the inquired behavior. Further studies should investigate whether the PAS can be appropriately used for 2-year olds as well. For the age of 4 and 5, values of the German children seem to get closest to the Australian ones. The group of the 6-year olds is too small to reasonably compare it to 6-year olds of other countries.

It should be a topic of further research to investigate in a longitudinal study whether the mean scores change over time. As studies have shown that anxiety disorders seem to persist over the course of time [18–21] it may be interesting whether the total score of anxious or less anxious children remains steady while the content of fears can change with age and cognitive development.

The present study supports the core structure of fears: they cluster into five correlated factors representing the five scales separation anxiety, social anxiety, obsessive–compulsive disorder, generalized anxiety and physical injury fear even as early as in preschool age. As in other PAS studies [28, 35, 40, 41] we found the five factors to be strongly inter-correlated. The strong covariance was well explained by a higher-order factor representing a more general anxiety (vulnerability).

Due to the fact that the questionnaire is meant to be completed by the parents, biases may occur. This seems to be especially true for mothers suffering from depression or anxieties [71]. Further studies should therefore consider to assess mental health of the informant. Moreover, in the present study mother and father ratings were not analyzed separately, because the subsample of fathers was too small to allow for separate analyses. A recent study from Jansen, Bodden, Muris, van Doorn and Granic [72] has shown that there is a significant correlation between maternal and paternal ratings although mothers showed significantly more correspondence with their children. Nonetheless, children in the named study were on average already 10 years old. Further studies should evaluate whether this is also true for younger children by additionally including direct behavioral observations or anxiety ratings (by for example nursery school teachers).

## Summary

In this study, we analyzed the factor structure and psychometric properties of the Preschool Anxiety Scale [28] and used the questionnaire to investigate anxieties in a sample of German preschoolers. We additionally offered an overview of PAS-values from other countries to allow an approximate classification of the anxieties of German Preschoolers. Our study supports the results of other PAS studies that anxieties cluster into five correlated factors. Children living in foster care were perceived as significantly less socially anxious than children living in their biological families. Apart from that there was no significant difference between the two groups. About 17% of the children living in their biological families and 19% (21% when leaving out the SA scale) of the children living in foster care “would warrant further investigation”. These results fit well with the prevalence rates of anxieties in preschool children reported by previous studies. Regarding the content, German preschoolers displayed the same anxieties most common among preschoolers in other countries. Nonetheless, anxieties in preschoolers seem to be reported more seldom by German parents compared to parents from other countries. Additional research is needed to identify potential reasons for these cross-country differences. Economic hardship may be one among others.

## Supplementary Information

Below is the link to the electronic supplementary material.

Supplementary file1 (DOCX 12 kb)

Supplementary file2 (DOCX 14 kb)

Supplementary file3 (DOCX 14 kb)

Supplementary file4 (DOCX 14 kb)

Supplementary file5 (DOCX 13 kb)

Supplementary file6 (DOCX 11 kb)

## Footnotes

1 For one child only one half of the questionnaire was completed. It can be assumed that the related parent just did not see the backside of the questionnaire. The missings can therefore be considered as being not random. The remaining four children were at the age of two or had just turned three. It is noticeable that parents only answered those questions referring to directly observable behavior. It can be assumed that those parents did not feel able to evaluate internal psychological processes of their children because they were probably too young to talk about their feelings. These missings were therefore considered as not being random as well.
