# Supplementary material for: How Anxious are German Preschool Children?
Source: Child Psychiatry Hum Dev. 2021 May 8;53(5):992–1003. doi: 10.1007/s10578-021-01185-8 (PMC9470646; doi:10.1007/s10578-021-01185-8)
Supplement: Supplementary file 1 — Supplementary file1 (DOCX 12 kb) [file 10578_2021_1185_MOESM1_ESM.docx]

| Table E-1  *Minimum and maximum value (range), mean value (M), median (Med), modus (Mod), variance (V), standard deviation (SD), span (Sp), skewness and kurtosis for the total score and all subscales, N=577* | | | | | | | | | |
| --- | --- | --- | --- | --- | --- | --- | --- | --- | --- |
|  | Range | M | Med | Mod | V | SD | Sp | skewness | kurtosis |
| TS | 0 - 112 | 14.94 | 13.00 | 9.00 | 113.18 | 10.64 | 75 | 1.38 | 3.70 |
| GAD | 0 - 20 | 1.78 | 1.00 | 0.00 | 5.58 | 2.36 | 18 | 2.36 | 8.73 |
| SA | 0 - 24 | 3.87 | 3.00 | 0.00 | 13.08 | 3.62 | 22 | 1.24 | 1.94 |
| OCD | 0 - 20 | 1.13 | 0.00 | 0.00 | 3.41 | 1.85 | 13 | 2.72 | 9.99 |
| PIF | 0 - 28 | 5.12 | 4.00 | 4.00 | 5.12 | 3.77 | 24 | 0.95 | 1.58 |
| SAD | 0 - 20 | 3.04 | 3.00 | 0.00 | 8.26 | 2.87 | 16 | 1.20 | 1.80 |
| *Note.* Standard error of the skewness = 0.10, standard error of the kurtosis = 0.20; TS = total score of the PAS, GAD = generalized anxiety disorder, SA = social anxiety, OCD = obsessive-compulsive disorder, PIF = physical injury fears, SAD = separation anxiety disorder; To determine whether skewness and kurtosis show a significant deviation from a normal distribution fisher skewness coefficient (Sk_F_) and fisher coefficient of kurtosis (Ku_F_) was calculated (Pett, 2016). All calculated values exceeding the range of ± 1.96 can be seen as significant. | | | | | | | | | |
