# Supplementary material for: How Anxious are German Preschool Children?
Source: Child Psychiatry Hum Dev. 2021 May 8;53(5):992–1003. doi: 10.1007/s10578-021-01185-8 (PMC9470646; doi:10.1007/s10578-021-01185-8)
Supplement: Supplementary file 2 — Supplementary file2 (DOCX 14 kb) [file 10578_2021_1185_MOESM2_ESM.docx]

| Table E-2  *Means, standard deviation, internal consistency and p-values for child anxiety separated by biological and foster parents and by gender* | | | | | | | | | | | | | | | | |  |
| --- | --- | --- | --- | --- | --- | --- | --- | --- | --- | --- | --- | --- | --- | --- | --- | --- | --- |
|  | Biological Parents | | | | | | |  |  | Children in foster care | | | | | | |  |
|  | Boys^1^ | | |  | Girls^2^ | | |  |  | Boys^3^ | | |  | Girls^4^ | | |  |
|  | *M* | *SD* | *α* |  | *M* | *SD* | *α* | *p* |  | *M* | *SD* | *α* |  | *M* | *SD* | *α* | *p* |
| TS (28 items) | 14.02 | 9.78 | .836 |  | 16.00 | 10.86 | .864 | .034* |  | 14.09 | 11.89 | .884 |  | 15.35 | 12.50 | .876 | .629 |
| GA (5 items) | 1.58 | 2.01 | .680 |  | 1.84 | 2.47 | .784 | .193 |  | 1.89 | 2.22 | .595 |  | 2.49 | 3.47 | .798 | .334 |
| SA (6 items) | 3.83 | 3.68 | .787 |  | 4.26 | 3.63 | .772 | .197 |  | 2.73 | 2.92 | .687 |  | 3.14 | 3.52 | .800 | .556 |
| OCD (5 items) | 1.07 | 1.83 | .452 |  | 1.04 | 1.74 | .518 | .871 |  | 1.56 | 2.21 | .406 |  | 1.51 | 2.07 | .319 | .924 |
| PiF (7 items) | 4.75 | 3.56 | .523 |  | 5.57 | 3.89 | .607 | .016* |  | 5.24 | 4.36 | .647 |  | 4.72 | 3.40 | .355 | .553 |
| SAD (5 items) | 2.79 | 2.44 | .461 |  | 3.29 | 3.03 | .629 | .045* |  | 2.67 | 2.98 | .691 |  | 3.49 | 3.95 | .751 | .272 |

*Note.* ^1^*N* = 250, ^2^*N* = 239, ^3^*N* = 45, ^4^*N* = 43; * significant *p*-value (<.05); TS = total score of the PAS, GA = generalized anxiety, SA = social anxiety, OCD = obsessive-compulsive disorder, PiF = physical injury fears, SAD = separation anxiety disorder
