# Supplementary material for: How Anxious are German Preschool Children?
Source: Child Psychiatry Hum Dev. 2021 May 8;53(5):992–1003. doi: 10.1007/s10578-021-01185-8 (PMC9470646; doi:10.1007/s10578-021-01185-8)
Supplement: Supplementary file 3 — Supplementary file3 (DOCX 14 kb) [file 10578_2021_1185_MOESM3_ESM.docx]

| Table E-3  *Rank order percentage of children receiving parent ratings of 3 or 4 (Quite Often True and Very Often True) for each item for biological (BF) and children raised in foster care (FC)* | | | |
| --- | --- | --- | --- |
|  |  | % of ratings ≥ 3 | |
|  | PAS Items | BF | FC |
| 6. | Is reluctant to go to sleep without you or to sleep away from home | 20.5 | 20.4 |
| 20. | Is afraid of insects and/or spiders | 12.1 | 17.0 |
| 13. | Is scared of thunder storms | 10.6 | 10.2 |
| 11. | Is afraid of meeting or talking to unfamiliar people | 10.5 | 7.9 |
| 24. | Is frightened of dogs | 10.2 | 4.5 |
| 26. | Is afraid of the dark | 9.4 | 18.2 |
| 23. | If afraid to go up to group of children and join their activities | 5.1 | 2.2 |
| 15. | Is afraid of talking in front of the class (preschool group) e.g., show and tell | 4.5 | 1.1 |
| 2. | Worries that he/she will do something to look stupid in front of other people | 3.7 | 1.1 |
| 22. | Becomes distressed about your leaving him/ her at preschool/school or with a babysitter | 3.5 | 4.5 |
| 1. | Has difficulty stopping him/herself from worrying | 3.5 | 3.4 |
| 3. | Keeps checking that he/she has done things right (e.g., that he/she closed a door, turned off a tap) | 3.4 | 2.3 |
| 12. | Worries that something bad will happen to his/her parents | 3.2 | 1.1 |
| 17. | Is nervous of going swimming | 3.0 | 6.8 |
| 9. | Washes his/her hands over and over many times each day | 2.6 | 3.4 |
| 5. | Is scared to ask an adult for help (e.g., a preschool or school teacher) | 2.4 | 3.4 |
| 7. | Is scared of heights (high places) | 2.2 | 3.4 |
| 16. | Worries that something bad might happen to him/her (e.g., getting lost or kidnapped), so he/she won’t be able to see you again | 2.2 | 4.6 |
| 19. | Worries that he/she will do something embarrassing in front of other people | 2.2 | 1.1 |
| 8. | Has trouble sleeping due to worrying | 1.8 | 3.4 |
| 28. | Asks for reassurance when it doesn’t seem necessary | 1.6 | 5.7 |
| 10. | If afraid of crowded or closed-in places | 1.2 | 3.4 |
| 25. | Has nightmares about being apart from you | 1.2 | 5.7 |
| 4. | Is tense, restless or irritable due to worrying | 1.0 | 3.4 |
| 18. | Has to have things in exactly the right order or position to stop bad things from happening | 0.8 | 4.6 |
| 21. | Has bad or silly thoughts or images that keep coming back over and over | 0.6 | 2.2 |
| 14. | Spends a large part of each day worrying about various things | 0.2 | 1.1 |
| 27. | Has to keep thinking special thoughts (e.g., numbers or words) to stop bad things from happening | 0.0 | 0.0 |

*Note.* BF = children raised in their biological families, *N* = 489; FC = children raised in foster care, *N* = 88
