# Supplementary material for: How Anxious are German Preschool Children?
Source: Child Psychiatry Hum Dev. 2021 May 8;53(5):992–1003. doi: 10.1007/s10578-021-01185-8 (PMC9470646; doi:10.1007/s10578-021-01185-8)
Supplement: Supplementary file 4 — Supplementary file4 (DOCX 14 kb) [file 10578_2021_1185_MOESM4_ESM.docx]

| Table E-4  *Effect sizes for the comparison between Germany and the countries mentioned below for every PAS-scale separated by age group* | | | | | | | | | | | | | | | |
| --- | --- | --- | --- | --- | --- | --- | --- | --- | --- | --- | --- | --- | --- | --- | --- |
| age | 3 | | |  | 4 | | |  | 2-4 |  | 5 | | |  | 5-6 |
|  | Aus. | Rom. | Port. |  | Aus. | Rom. | Port. |  | Neth. |  | Aus. | Rom. | Port. |  | Neth. |
| N | 270 | 118 | ? |  | 402 | 200 | ? |  | 137 |  | 551 | 236 | ? |  | 135 |
| TS | 0.59 | 1.18 | 1.17 |  | 0.30 | 1.29 | 1.18 |  | 0.36 |  | 0.12 | 1.00 | 1.25 |  | 0.25 |
| GAD | 0.47 | 0.91 | 1.13 |  | 0.39 | 0.98 | 1.26 |  | 0.43 |  | 0.18 | 0.61 | 1.07 |  | 0.40 |
| SA | 0.46 | 0.38 | 0.74 |  | 0.26 | 0.53 | 0.60 |  | 0.43 |  | 0.18 | 0.61 | 1.07 |  | 0.40 |
| OCD | 0.34 | 1.52 | 1.05 |  | 0.26 | 0.53 | 0.60 |  | 0.27 |  | 0.17 | 0.33 | 0.75 |  | 0.35 |
| PiF | 0.65 | 0.77 | 0.58 |  | 0.32 | 0.96 | 0.78 |  | 0.48 |  | 0.07 | 1.14 | 0.94 |  | 0.24 |
| SAD | 0.24 | 0.99 | 0.87 |  | 0.03 | 0.95 | 0.94 |  | -0.27 |  | -0.14 | 0.68 | 1.05 |  | -0.29 |

*Note.* Aus. = Australia, Spence et al. (2001), Rum. = Rumanian, Benga et al. (2010), Neth. = Netherlands Broeren & Muris (2008), Port. = Portugal, Almeida & Viana (2013). In the Portuguese sample it is not given how the number of children spreads across the age groups (*N* = 562, *M* = 51.19 month, *SD* = 13.39). TS = total score of the PAS, GAD = generalized anxiety disorder, SA = social anxiety, OCD = obsessive-compulsive disorder, PIF = physical injury fears, SAD = separation anxiety disorder
