# Supplementary material for: How Anxious are German Preschool Children?
Source: Child Psychiatry Hum Dev. 2021 May 8;53(5):992–1003. doi: 10.1007/s10578-021-01185-8 (PMC9470646; doi:10.1007/s10578-021-01185-8)
Supplement: Supplementary file 5 — Supplementary file5 (DOCX 13 kb) [file 10578_2021_1185_MOESM5_ESM.docx]

| Table E-5  *Factor loadings for the five-correlated factor model* | | | | | | |
| --- | --- | --- | --- | --- | --- | --- |
| Predicted DSM-IV category | Questionnaire items | Factor loadings | | | | |
|  |  | F1 | F2 | F3 | F4 | F5 |
| Generalized anxiety | 1. difficult stop worrying | .67 |  |  |  |  |
|  | 4. tense etc. due worrying | .74 |  |  |  |  |
|  | 8. trouble sleep due worrying | .63 |  |  |  |  |
|  | 14. large part day worrying | .70 |  |  |  |  |
|  | 28. asks reassurance not neces. | .42 |  |  |  |  |
| Social anxiety | 2. worries look stupid front others |  | .61 |  |  |  |
|  | 5. scared ask adult for help |  | .60 |  |  |  |
|  | 11. afraid unfamiliar people |  | .60 |  |  |  |
|  | 15. afraid talking front class group |  | .67 |  |  |  |
|  | 19. worries do embarrassing fr. o. |  | .59 |  |  |  |
|  | 23. afraid join group activities |  | .62 |  |  |  |
| OCD | 3. checking done things right |  |  | .51 |  |  |
|  | 9. washes hands many times day |  |  | .36 |  |  |
|  | 21. silly thoughts come back |  |  | .56 |  |  |
|  | 18. do things in the right order stop bad |  |  | .54 |  |  |
|  | 27. think special thoughts stop bad |  |  | .38 |  |  |
| Physical injury fear | 7.  scared of heights |  |  |  | .42 |  |
|  | 10. afraid of crowded places |  |  |  | .55 |  |
|  | 13. nervous thunderstorms |  |  |  | .44 |  |
|  | 17. nervous going swimming |  |  |  | .40 |  |
|  | 20. afraid of insects |  |  |  | .40 |  |
|  | 24. frightened of dogs |  |  |  | .32 |  |
|  | 26. afraid of the dark |  |  |  | .50 |  |
| Separation anxiety | 6. reluctant go sleep alone |  |  |  |  | .34 |
|  | 12. worries bad happen to parents |  |  |  |  | .53 |
|  | 16. worries bad happen to his/her |  |  |  |  | .69 |
|  | 22. distressed about p. leaving h/h |  |  |  |  | .49 |
|  | 25. nightmares being apart of p. |  |  |  |  | .60 |
