# Supplementary material for: How Anxious are German Preschool Children?
Source: Child Psychiatry Hum Dev. 2021 May 8;53(5):992–1003. doi: 10.1007/s10578-021-01185-8 (PMC9470646; doi:10.1007/s10578-021-01185-8)
Supplement: Supplementary file 6 — Supplementary file6 (DOCX 11 kb) [file 10578_2021_1185_MOESM6_ESM.docx]

| Table E-6  *Factor intercorrelations for five-factor model* | | | | | |
| --- | --- | --- | --- | --- | --- |
| Factor | GA | SA | OCD | PiF | SA |
| GA | 1.00 | - | - | - | - |
| SA | .51 | 1.00 | - | - | - |
| OCD | .90 | .46 | 1.00 | - | - |
| PiF | .58 | .51 | .67 | 1.00 | - |
| SA | .81 | .51 | .84 | .79 | 1.00 |

*Note.* GAD = generalized anxiety disorder, SA = social anxiety, OCD = obsessive-compulsive disorder, PiF = physical injury fears, SAD = separation anxiety disorder
